# Anti-Müllerian Hormone in Pathogenesis, Diagnostic and Treatment of PCOS

**DOI:** 10.3390/ijms222212507

**Published:** 2021-11-19

**Authors:** Ewa Rudnicka, Michał Kunicki, Anna Calik-Ksepka, Katarzyna Suchta, Anna Duszewska, Katarzyna Smolarczyk, Roman Smolarczyk

**Affiliations:** 1 Department of Gynecological Endocrinology, Medical University of Warsaw, 00-315 Warsaw, Poland; ewa.rudnicka@poczta.onet.pl (E.R.); mkunicki@op.pl (M.K.); calikowna@wp.pl (A.C.-K.); suchta.katarzyna@gmail.com (K.S.); rsmolarczyk@poczta.onet.pl (R.S.); 2 Department of Morphological Sciences, Faculty of Veterinary Medicine, Warsaw University of Life Sciences, 02-776 Warsaw, Poland; duszewskaanna@hotmail.com; 3 Department of Dermatology and Venereology, Medical University of Warsaw, 02-008 Warsaw, Poland

**Keywords:** AMH, PCOS, hyperandrogenism

## Abstract

Polycystic ovary syndrome (PCOS) is the most common endocrine disorder among reproductive-aged women. It is characterized by chronic anovulation, hyperandrogenism, and the presence of polycystic ovary in ultrasound examination. PCOS is specified by an increased number of follicles at all growing stages, mainly seen in the preantral and small antral follicles and an increased serum level of Anti-Müllerian Hormone (AMH). Because of the strong correlation between circulating AMH levels and antral follicle count on ultrasound, Anti-Müllerian Hormone has been proposed as an alternative marker of ovulatory dysfunction in PCOS. However, the results from the current literature are not homogeneous, and the specific threshold of AMH in PCOS and PCOM is, therefore, very challenging. This review aims to update the current knowledge about AMH, the pathophysiology of AMH in the pathogenesis of PCOS, and the role of Anti-Müllerian Hormone in the treatment of this syndrome.

## 1. Introduction

Polycystic ovary syndrome (PCOS) is one of the most common endocrine disorders among reproductive-aged women and affects 10–15% of them [1,2]. The condition is heterogeneous, and women may present with reproductive, endocrine, and/or metabolic symptoms, which vary across their lifespan. The Rotterdam criteria requires women to fulfil two of the following three criteria to be diagnosed: (1) oligo-or anovulation, (2) clinical and/or biochemical signs of hyperandrogenism, and/or (3) polycystic ovaries on ultrasound, with the exclusion of other relevant disorders [3]. The pathogenesis of PCOS is not fully understood. One of the proposed mechanisms for hyperandrogenism is follicle maturation abnormalities, in which the growing follicle does not progress to a dominant follicle [4]. In women, AMH (Anti-Müllerian Hormone) inhibits the recruitment of primordial follicles out of the resting oocyte pool and may suppress follicle-stimulating hormone (FSH) action contributing to ovulatory disturbances [4,5]. AMH expression continues until follicles reach approximately 8 mm in diameter, and expression is deficient in larger antral follicles. Consequently, there is a good correlation between AMH and antral follicle count (AFC) [6,7,8]. Furthermore, women with PCOS have high AMH concentrations. Accordingly, AMH has been proposed as a marker of polycystic ovary syndrome and a substitute for AFC in diagnosing PCOS, especially when the ultrasound criteria remain controversial [7]. A variety of cutoff values of AMH have been proposed, but with varying sensitivity and specificity, the optimal threshold is unknown. The purpose of the present study is to explain the relationship between AMH and PCOS and to describe the importance and utility of serum AMH in diagnosing PCOS.

## 2. Physiology of AMH

Anti-Müllerian Hormone (AMH, also called Müllerian inhibiting substance-MIS) is a homodimeric glycoprotein belonging to the transforming growth factor-β (TGFβ) superfamily [4,9]. The AMH gene is located on the short arm of chromosome 19. AMH derives from the biologically inactive precursor proAMH. The active form of AMH consists of C-terminal that drives biological activity and N-terminal dimer [9]. Two types of AMHRI and AMHRII receptors and the SMAD proteins are involved in signal transmission by AMH. Usually, AMH binds to the AMH type II receptor (AMHR2) with high affinity through C-terminal [9], whereas SMAD proteins are unique transcription particles which transduce intracellular signaling to promote nuclear and physiological effects while binding AMH to its receptor [9,10].

AMH is released both in women, in the ovaries, and in men, in the testes. AMH plays a crucial role in gonadal sex differentiation prenatally by suppressing the development of the Müllerian ducts in males [9]. Testes produce AMH by immature Sertoli cells during embryonic development. Secretion of AMH starts from the eighth post-conception week until 2 years old postnatally, declining over the pubertal years and eventually being undetectable in adults [9,10], mainly due to high testosterone levels. If a male fetus has low AMH-levels or no AMH, this causes the simultaneous development of male and female genitalia.

In women, production starts from the 36th post-conception week. Then, after a transient neonatal peak, AMH levels remain low until puberty. Rising AMH serum levels to a plateau occurs in adolescent girls. Decline stars after the mid-20s and AMH serum levels eventually become undetectable several years before menopause [4,11]. Changes in AMH even precede modifications in FSH (follicle-stimulating hormone), inhibin B serum levels, and antral follicle count. AMH secretion drops dramatically before menopause due to the exhaustion of the follicle pool [10].

AMH is involved in developing ovarian follicles and influences the hypothalamic-pituitary-gonadal (HPG) axis at various stages of development [12]. Simultaneously, the regulation of AMH production may depend on many intracellular and extracellular signals. One of these may be from oocytes, which stimulate AMH secretion by releasing growth differentiation factor 9 (GDF9) and bone morphogenetic factor 15 (BMP15). Interestingly, FSH opposes the effects of GDF9 and BMP15 and thus prevents AMH overexpression [9].

AMH is released by granulosa cells and theca cells already in the primordial follicle, albeit at low levels. A much higher expression of AMH, which is possible to measure in the blood, is observed in preantral and small antral follicles with a 2–4 mm diameter [13]. AMH expression is not observed in large antral follicles, although its trace expression is observed in a preovulatory follicle, though this is not possible to measure in the blood [11,12,13,14]. The role of AMH in folliculogenesis is based on inhibition of primary follicle recruitment through its paracrine effect and inhibition of selection from a pool of small antral follicles through gonadotropin recruitment [4,9].

The serum AMH concentration reflects the ovarian follicle pool with a strong dependence on the number of antral follicles in the early follicular phase of the menstrual cycle [12,13,14]. Therefore, a low antral follicle count may result in low serum AMH levels. Moreover, the AMH blood level does not depend on the menstrual cycle phase or exogenous sex steroids. Therefore, blood samples can be taken on any day of the cycle [4]. It should be emphasized that AMH is a recognized biomarker of female reproductive potential, reflecting the number of primary follicles and their response to exogenous gonadotrophins [1,12,14].

## 3. AMH Assay

There are many difficulties and technical problems in AMH serum measurement [15]. The difficulty lies in the fact that there are currently various ELISA immunoassays available worldwide: the Gen II (Beckam Coulter, Pasadena, CA, USA), the EIA AMH/MHS kits (IOTor, Immunotech Beckman Coulter) and the AL-105-I (Anshlabs, San Francisco, CA, USA), which use different monoclonal antibodies and different standards [16]. To improve the clinical utility of serum AMH measurement, new methods have been introduced: the development of an ultrasensitive assay (pico AMH kit, Anshlabs, San Francisco, CA, USA), and automation on immuno-analyzers (Access Dxi automatic analyzer, Beckman Coulter. Cobas e instrument, Roche Diagnostics, Basel, Switzerland), allowing nearly identical values [17]. The second problem with AMH assay is the molecular heterogeneity of the circulating AMH level with a non-cleaved biologically active and nonactive form, the not well-known stability of AMH samples during storage, and the variable sensitivity of the immunoassays to interference by complement C1q and C3 [18]. Otherwise, the AMH concentration varies according to the situation; in male newborns, they are very high (749–1930 pmol/L), and in adults, they are low (10.7–98 pmol/L). Therefore, measurement involves different assays with different sensitivities [15].

## 4. AMH as an Indicator of Ovarian Reserve and Follicle Growth

In women, Anti-Müllerian Hormone (AMH) is expressed by growing follicles of up to 8 mm [4]. AMH is secreted before FSH-dependent selection of dominant follicle; therefore, it is independent of FSH impact. AMH may also protect growing follicles from premature maturation. This is possible due to its opposing action to the effects of FSH.

Many studies indicate the correlation between serum AMH level and the number of growing follicles. In addition, both AMH serum level and the number of follicles decrease with the aging of women [12,14,19]. Based on the observations mentioned above, AMH is regarded as an indirect marker of ovarian reserve in women. However, it is worth noting that AMH is expressed only by growing follicles, so its serum concentration does not correlate with the number of primordial follicles among young women. Considering this fact, it can be said that AMH serum level reflects functional ovarian reserve (FOR). FOR consists of cohorts of small growing follicles from 2 mm to 5 mm from which FSH will choose the dominant follicle to ovulate [20,21]. The situation is different in older women. Some studies indicate that among women of late reproductive age, serum AMH level, apart from FOR, can be helpful in assessing primordial follicle density [22,23,24,25]. When using AMH serum concentration to evaluate FOR, the results of the latest studies should be taken into consideration. It has recently been shown that intracycle variation up to 20% of serum AMH level can be observed [26,27,28].

Furthermore, some studies also showed an intercycle variation of AMH serum level between 28 and 163% depending on the AMH assay used [26,29]. Therefore, it seems reasonable to make therapeutic decisions based on repeated assessments of AMH serum levels using the same assay. Apart from evaluating FOR by AMH serum level concentrations, it is still unclear whether AMH levels can play a predictive role in assessing spontaneous fertility. Data remain unclear [30,31,32]. Several factors that influence it should be considered to give the appropriate meaning of AMH serum levels in assessing ovarian reserve.

Hormonal contraception is a factor that influences AMH serum level. AMH serum level is lower and varies from 14 to 55% among women who use hormonal contraception [33]. In addition, a retrospective study by Landersoe showed that variation in AMH serum level among women who use hormonal contraception depends on the method used. For example, women using oral contraceptives or progesterone-only pills have 30 to 40% lower AMH serum levels. In comparison, women using intrauterine devices have 17% lower AMH serum levels [34]. Some studies indicate the impact of body mass index (BMI) on AMH serum levels. For example, Moslehi, based on 26 studies, showed a negative correlation between BMI and AMH serum levels [35].

Recent studies also showed an impact of vitamin D serum concentration on AMH serum levels. Both vitamin D and AMH present seasonal serum variability during the year, with higher serum concentration during the summer and lower serum concentration during the winter. It was indicated that higher vitamin D serum concentration results in higher AMH serum levels [36,37,38].

## 5. AMH in Pathogenesis and Treatment of PCOS

### 5.1. Pathophysiology

**Figure 1:**
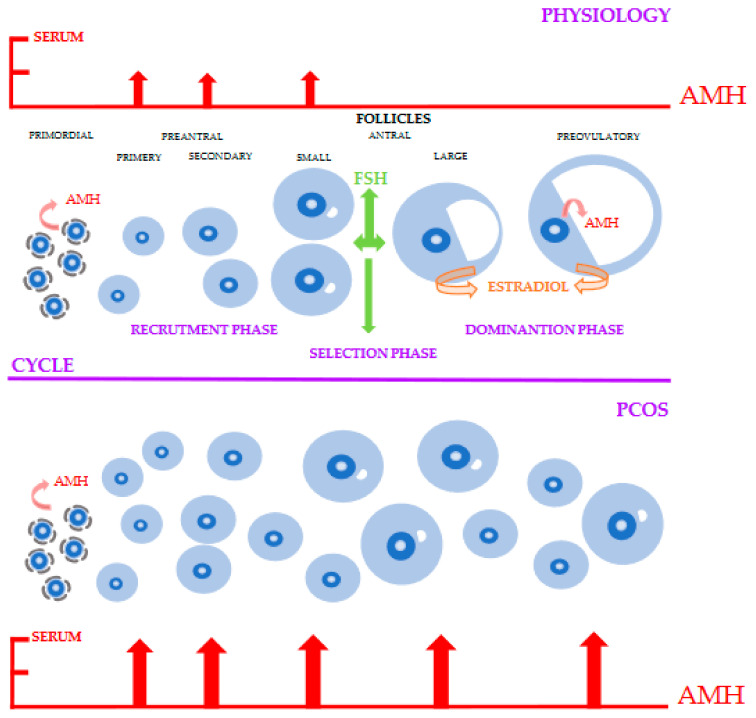
The roles of AMH (Anti-Müllerian Hormone) in PCOS (Polycystic Ovary Syndrome). In the physiological cycle, during the FSH (Follicle Stimulating Hormone) independent phase, AMH expression is lower in activated primordial follicles and highest in preantral and small antral follicles. In the FSH dependent phase, the expression of AMH is blocked. However, lower expression of AMH is observed in the preovulatory follicle. Therefore, the absence of AMH leads to an increased Estradiol level in antral and preovulatory follicles. In PCOS, the expression of AMH increases twofold, leading to an increased number of recruited follicles. In addition, such a high level of AMH reduces FSH expression and Estradiol synthesis, thereby blocking the selection phase and thus the formation of a preovulatory follicle.

Intrafollicular and serum AMH levels are elevated in patients with PCOS–Figure 1 [39,40].

The elevated AMH level is 2–4-fold higher in women with PCOS than in healthy women and was described in all PCOS populations [16,41,42]. This increase is mainly seen in the preantral and small antral follicles and is caused by the increased number of small follicles and an increased secretion within each of these [16]. In the beginning, it was thought that the increase in AMH concentration in PCOS women was only due to the higher number of preantral and small antral follicles. However, the production of AMH was detected in vitro 75-fold higher in anovulatory PCOS and 20-fold higher in normal-ovulatory PCOS than in healthy controls [16]. The cause of such overproduction of AMH is still unknown, but there is evidence determining the role of androgens. A positive correlation between serum androgens and AMH concentration was found [43,44]. It is also due to intrinsic dysregulation of the granulosa cells, in which an increase in AMH receptor type II was described [45,46,47]. This excess of AMH is strongly suspected of playing a central role in the characteristic follicular arrest of PCOS, through the adverse action on aromatase expression and on FSH action [44]. For some authors, gonadotropins (especially FSH) inhibit serum AMH production. On the other hand, it described a stimulating effect of FSH on AMH expression in normal and polycystic ovaries [47,48]. This controversial point could be reconciled by recent findings that E2 inhibits AMH expression mediated via estrogen receptor ß [49]. In small antral follicles, FSH could directly stimulate AMH, but by increasing E2 production in larger follicles, FSH may inhibit AMH expression through the negative feedback of E2 [49]. This mechanism is disrupted in PCOS women, for whom the simultaneous increase in E2 levels was absent. For this reason, AMH expression and follicular fluid AMH levels decline in gonadotropin-dependent follicles in normal ovulatory women, but not in PCOS patients [50]. Clinical studies also showed a stimulating role of LH on AMH production [51]. Both theca and granulosa cells of small antral follicles express higher luteinizing hormone (LH) receptor levels in PCOS women compared to normal ovulatory women [52]. Combined with the elevated LH levels in PCOS, this leads to hyperstimulation of the theca cells and premature luteinization of granulosa cells. Furthermore, there are also data that LH stimulation increased AMH expression in granulosa cells of PCOS women but not in normal ovulatory women [5,46,47].

Recent studies also indicate that AMH may play a role in the neuroendocrine dysregulation in PCOS [53,54]. This hypothesis is based on detecting AMHR2 expression in hypothalamic GnRH neurons in both rodents and humans [54]. These neurons are AMH responsive because AMH stimulated the release and excitability of GnRH in neuronal explants of rats. Furthermore, AMH injected directly into the lateral ventricle of female mice induced a rapid increase in GnRH-mediated LH pulsatility and secretion [54]. In PCOS patients, AMH concentration is positively correlated with LH levels, and high LH stimulates the release of ovarian androgens production by theca cells. This suggests the existence of a positive feedback loop between AMH, GnRH and LH in PCOS [12,55].

It is also worth highlighting the role of AMH in placental function in PCOS. Recently, placental AMHR2 expression has been detected in various species, including humans [56,57]. Furthermore, a study by Tata et al. [57] investigated whether daily injection of AMH during E16.5–E18.5 of pregnancy surprised significantly placental Cyp19a1 expression. Placental aromatization is essential to protect the fetus from virilization by the fetal androgens and to prevent the accumulation of high androgen levels in the maternal circulation. Interestingly, AMH declines in pregnant normal ovulatory women after the first trimester, while in PCOS lean women, AMH levels remain significantly higher during pregnancy [57,58]. Thus, elevated AMH concentration may lead to a hyperandrogenic environment, which could reprogram the reproductive axis of female offspring [57].

### 5.2. AMH in Diagnosis and Treatment of PCOS

Because of a strong correlation between circulating AMH levels and antral follicle count on ultrasound, Anti-Müllerian Hormone has been proposed as an alternative maker of ovulatory dysfunction in PCOS or even as a single test for the diagnosis of PCOS [6,19,59]. However, the results from the current literature are not homogeneous, and the specific threshold of AMH in PCOS and PCOM is therefore very challenging [8,59,60]. Heterogeneity between researchers relates to assays, life stages, and phenotypes studied. Some authors have used the PCOS definition from the 2003 Rotterdam criteria, using 12 follicles of 2–9 mm diameter per ovary for the polycystic ovaries morphology (PCOM) [59]. Therefore, the threshold has evolved with the latest ultrasound generation, and it is now up to 19 or 25 follicles [61,62].

Additionally, there are technical issues in AMH assays leading to the heterogeneity of the results and the differences that arise from the population who is included in or excluded from the research. For these reasons, serum AMH levels should not be used as an alternative or a single test for PCOS diagnosis. To date, a cutoff at 35 pmol/L (4.9 ng/mL) with the enzyme immunoassay (AMH-EIA) provided by Beckam Coulter is the most proposed cutoff point with reasonable specificity (97%) and a better sensitivity than the AFC (antral follicle count) (92%), to distinguish women with PCOS from healthy women [19].

Serum AMH concentration is correlated with the severity of PCOS symptoms. AMH levels were significantly higher when hyperandrogenism was present, and it has been proposed that a high serum AMH concentration can be considered a marker of hyperandrogenism and can be used as a substitute for this status in the Rotterdam criteria [16,63]. One of the underlying pathophysiologies of PCOS is insulin resistance. There are no clear correlations between levels of AMH with the incidence of insulin resistance in PCOS patients. A significant positive correlation between AMH and HOMA-IR (Homeostatic Model Assessment for Insulin Resistance) was observed in the study by Wiweko [64] and Skałba [65]. However, a negative correlation or no correlation have also been reported. According to some studies, AMH serum levels could also be used as a prognostic factor of metformin therapy in clinical practice. In the work of Tomova et al., AMH value decreases after treatment with metformin [66]. It is prevalent to prescribe hormonal contraception, sometimes with antiandrogens, for women with PCOS syndrome to normalize the menstrual cycle and treat acne and hirsutism. Pandis et al. reported a significant decrease in serum AMH concentration in women with PCOS treated with ethinylestradiol (EE) + cyproterone acetate (CPA) but not with EE + drospirenone [67]. Some investigations studied the relationship between weight loss, menstrual cycles, and AMH concentrations in overweight women with PCOS. Moran et al. and Nybacka et al. described a significant decrease in serum AMH levels after diet and weight loss [68,69].

Serum AMH levels are elevated in adolescents with PCOS [70,71]. They also have a higher antral follicle count and a larger ovarian size than adolescents without PCOS. Therefore, it is sometimes difficult to estimate the ovaries on ultrasonography in this group. In light of the difficulties and uncertainties associated with ovarian ultrasound in the adolescent population with PCOS, AMH may potentially replace its use for the diagnosis in this age group, which is also recommended by the American Association of Clinical Endocrinologists [72]. Although serum AMH levels cannot be used independently, they may be helpful as a part of an algorithm, along with clinical signs, androgen levels, and ultrasound [71].

### 5.3. AMH and Ovarian Response in Women with PCOS

Comprehensive literature shows that serum AMH can predict ovarian response to clomifene citrate (CC) or gonadotropins treatment in women with PCOS. When analyzing available literature, it is essential to note that the studies cover different populations, apply different AMH kits, and have different androgen concentrations and body mass index (BMI). These and other factors such as small sample size and retrospective design of the studies could influence the results [73,74,75,76,77,78,79,80,81]. It has been demonstrated that serum AMH levels are positively correlated with an antral follicular count, free testosterone, and luteinizing hormone levels. The severity of PCOS is associated with an increasing number of small follicles which produce AMH, and in turn, AMH has an impact on CC/gonadotropins response. The high AMH concentration could influence the sensitivity of follicles to gonadotropin stimulation. Most studies indicate the usefulness of AMH measurement in predicting CC response [74,75,76,77,78,79].

The high AMH pretreatment serum concentrations have diminished the chances of a response. In the case of a higher concentration of AMH in serum, higher doses of gonadotropins and extended treatment is recommended.

The data were also performed on different participants, including 20 nonobese anovulatory women with PCOS as in Amer’s study as well as 164 and 150 women in Sachdeva and Ellakwa, respectively [73,75,76]. In all studies mentioned above, the clomifene citrate was given with 50–150 mg/d. The data indicated that ovulation occurred in women with lower AMH serum concentrations. Thus, there was no one value of AMH above which CC-resistance could occur. It is also worth pointing out that according to receiver operation curve (ROC) analysis, specificity and sensitivity of the thresholds were different and not consistently high. The opposite conclusions were presented by Viarelli [80] El-Halawaty [81]. The former performed a retrospective study on 84 PCOS women who underwent CC stimulation followed by natural intercourse or intrauterine inseminations. They found that AMH measured with either Immunotech assay (IoT) or Gen II kits appeared to demonstrate minimal ability to identify PCOS patients who respond to 50 mg CC.

The latter found higher AMH in women who ovulated but compromised a subgroup of obese PCOS women treating a high dose of CC. Laparoscopic ovarian drilling (LOD) is one of the strategies applied in some groups of PCOS infertility patients. It is established that LOD could be an alternative to ovulation induction with gonadotropins for those who do not respond to clomiphene. This technique avoids multiple pregnancies related to gonadotropin treatments and provides a comparable pregnancy rate [82]. Studies have shown that preoperative high AMH concentrations could indicate the failure of induction of ovulation after LOD. According to some studies, the threshold varies from 7.7 ng/mL to 8.5 ng/mL [83,84] AMH in PCOS women undergoing in vitro fertilization (IVF) procedure. Numerous studies indicate the role of both low and high AMH in predicting IVF results [85,86,87,88]. Women with high AMH do not all fulfil PCOS criteria. The data regarding the prediction of serum AMH levels in women with diagnosed PCOS undergoing IVF procedures are inconsistent [89,90,91,92,93,94]. Silbersteinet [88] showed a correlation between AMH and embryo quality and stated that AMH higher than 2.7 ng/mL indicated a higher implantation rate (IR) and clinical pregnancy rate (CPR). Similar conclusions were drawn by Kaya [89] who found a strong association between serum AMH on day 3 of the stimulation cycle and the IR and CPR. A cutoff value of AMH was set as 3.2 ng/mL for predicting CPR, with a sensitivity of 72.7% and specificity of 77.3%. There is also no single threshold indicated on hyper response during IVF. The data predicted hyperresponsive with the cutoff value of 2.84 ng/mL, and the sensitivity and specificity were 72.7% and 65.9%, respectively, but also of 6.85 ng/mL with a sensitivity of 66.7% and a specificity of 68.7%.

## 6. Conclusions

Serum AMH is significantly higher in patients with PCOS than healthy controls, and it is now undeniable that AMH is a valuable tool for diagnosing PCOS. AMH level can be helpful, mainly if it is difficult to evaluate the ovaries during ultrasound examination and the AFC cannot be measured as in obese, virgin and poorly echogenic patients. AMH may also provide the pathogenesis of PCOS and the different phenotypes. However, a specific threshold for AMH concentration is very challenging, and according to the present state of knowledge, the level of AMH in serum should not be used as an alternative for detecting PCOM or as a single test for the diagnosis of PCOS. Nevertheless, it is very probable that with improved standardization of assays and established cut off levels, AMH may become an accurate diagnostic test for PCOS.

### Review Criteria

A search for original articles published between 1998 and 2021, focusing on: AMH in PCOS was performed in Scopus, Web of Science, PubMed. The search terms used were: “AMH”, “PCOS”, “Hyperandrogenism”, “AMH assay”, “ovarian reserve”, “AMH in PCOS”, “AMH in diagnosis and treatment of PCOS” The resulting references, including reviews, were used as leads for further literature searches.

## Data Availability

Not applicable.

## Abbreviations

AFC	Antral follicle count
AMH	Anti-Müllerian Hormone
AMH-EIA	AMH enzyme immunoassay
AMHR2	AMH type II receptor
BMI	body mass index
BMP15	bone morphogenetic factor 15
CC	clomifen citrate
CPA	cyproterone acetate
CPR	clinical pregnancy rate
EE	ethinylestardiol
FOR	functional ovarian reserve
FSH	Follicle stimulating hormone
GDF9	Growth differentiation factor 9
HOMA-IR	Homeostatic Model Assessment for Insulin Resistance
HPG	Hypothalamic–Pituitary–Gonadal
IOT	Immunotech assay
IR	implantation rate
IVF	in vitro fertilization
LOD	laparoscopic ovarian drilling
MIS	Müllerian inhibiting substance
PCOM	polycystic ovaries morphology
ROC	receiver operation curve
SMAD	proteins
TGF-B	transforming growth factor-B
VNO	vomeronasal organ
